# Supplementary material for: Moral Distress and Emotional Exhaustion in Healthcare Professionals: A Systematic Review and Meta-Analysis
Source: Healthcare (Basel). 2025 Feb 12;13(4):393. doi: 10.3390/healthcare13040393 (PMC11855070; doi:10.3390/healthcare13040393)
Supplement: Supplementary file 1 [file healthcare-13-00393-s001.zip › table 1 supplementary quality analysis.pdf]

Supplementary Table 1 - Quality of the studies and risk of bias assessment using the Q-SSP tool

Authors	*Scoring*	*Overall Quality Score (%)*
Christodolou et al. (2017)	16/19	84.21%
Delfrate et al. (2018)	14/19	73.64%
Doherty et al. (2022)	15/19	78.94%
Fumis et al. (2017)	17/19	89.74%
Grasso et al. (2022)	16/19	84.21%
Kellish et al. (2021)	17/19	89.74%
Kok et al. (2023)	16/19	84.21%
Malfoni et al. (2020)	17/19	89.74%
Maunder et al. (2023)	15/19	78.94%
Meltzer et al. (2004)	15/19	78.94%
Nassehi et al. (2023)	16/19	84.21%
Ohnishi et al. (2010)	16/19	84.21%
Rushton et al. (2015)	14/19	73.68%
Sajjadi et al. (2017)	16/20	80%
