# Supplementary material for: Moral Distress and Emotional Exhaustion in Healthcare Professionals: A Systematic Review and Meta-Analysis
Source: Healthcare (Basel). 2025 Feb 12;13(4):393. doi: 10.3390/healthcare13040393 (PMC11855070; doi:10.3390/healthcare13040393)
Supplement: Supplementary file 1 [file healthcare-13-00393-s001.zip › table 2 suplementary data extraction healthcare.pdf]

Table S2 – Characteristics of the included studies

Authors, year, country	*Design*	*Participants*	*Aim(s)*	*Data collection and scales*	*Analysis*	*Main results*
Maffoni et al. (2020)<br>Italy	Cross-sectional study	222 Italian professionals employed (nurses, physiotherapist, social health practitioner, physicians, psychologists) in neuro-rehabilitation medicine units.	To analyze whether managerial support and ethical vision of patient care would be related to emotional exhaustion or through moral distress.	Self-reported questionnaires<br><br>Moral Distress Scale-Revised (Hamric et al., 2012).<br>alpha = 0.87<br><br>Emotional Exhaustion. Five items from MBI (Maslach & Jackson, 1986).<br>alpha = 0.92	Descriptive statistics, correlations, mediation and moderated mediation models.	Moral distress increased emotional exhaustion. Therefore, moral distress partially mediated the relationship between ethical climate dimensions and emotional exhaustion.
Delfrate et al. (2018)<br>Italy	Cross-sectional study	228 psychiatric nurses employed in four public hospitals.	To assess the presence of moral distress among mental health nurses and verify whether there is a relationship between moral distress and emotional exhaustion.	Self reported questionnaires<br><br>Moral Distress Scale for Psychiatric Nurses (Ohnishi et al., 2010)<br>alpha = 0.93<br><br>Emotional Exhaustion from MBI (Maslach & Jackson, 1986)<br>alpha = 0.78	Descriptive statistics, correlations, Anova	Nurses working in Inpatients units had higher levels of moral distress than those working in Outpatients units and Rehabilitation units. Moral distress and emotional exhaustion showed a negative and significant relationship.
Rushton et al. (2015)<br>USA	Cross-sectional study	114 nurses in 6 high-intensity units: pediatric/neonatal, oncology, and adult critical care.	To explore factors involved in burnout, moral distress, and resilience.	Self-reported questionnaires<br><br>19-item version of the Moral Distress Scale (Corley et al., 2001)	Descriptive statistics, correlations, 1-way analysis of variance, multiple linear regression	Nurses working in high-risk areas, especially critical care, are at increased risk for burnout. The data showed a moderate correlation between moral distress and

				alpha = NR<br><br>Emotional Exhaustion from MBI (Maslach & Jackson, 1986)<br>alpha = NR	models	emotional exhaustion.
Maunder et al. (2023)<br>Canada	Six time-points cohort.	213 hospital workers (46 nurses, 71 other healthcare professionals, 34 non-professional staff with close patient contact, and 62 non-professional staff without close patient contact).	To assess contributors to moral distress and consequences of moral distress at a subsequent time in healthcare workers during the COVID-19 pandemic.	Self-reported questionnaires<br><br>Measure of Moral Distress for Healthcare Professionals (Epstein et al., 2019)<br>alpha = NR<br><br>Emotional Exhaustion from MBI (Maslach & Jackson, 1986)<br>alpha = 0.94	Descriptive statistics, chi-square tests, ordinal regression models, linear regression models.	The results supported a bidirectional relationship between moral distress and burnout (i.e. emotional exhaustion). Burnout both amplifies moral distress and occurs because of moral distress.
Carletto et al. (2022)<br>Italy	Cross-sectional study	115 healthcare providers (66.1% nurses and physiotherapists, 30.4% physicians 3.5% healthcare assistants).	To investigate the relationship between moral distress and burnout among neonatal intensive care unit (NICU) healthcare professionals.	Self-reported questionnaires<br><br>Italian version of the Moral Distress Scale-Revised (MDS-R) (Hanric et al., 2012)<br>alpha = NR<br><br>Emotional Exhaustion from MBI (Maslach & Jackson, 1986)<br>alpha = NR	Descriptive statistics, Mann-Whitney Utest, chi-square tests, linear regression models.	The results revealed a correlation between moral distress and burnout levels (i.e. emotional exhaustion). It was observed that the relationship between moral distress and emotional exhaustion was moderated by spirituality and/or religiousness.
Nassehi et al.	Cross-sectional	134 emergency	To explore the	Self-reported	Descriptive	A majority of EMTs

(2023)<br>Iran	study	medical technicians (EMT).	association between moral distress, burnout, and job satisfaction in emergency medical technicians during the COVID-19 pandemic.	questionnaires<br><br>Moral Distress Scale (Jafari et al., 2011)<br>alpha = 0.90<br><br>Emotional Exhaustion from MBI (Maslach & Jackson, 1986)<br>alpha = NR	statistics, t-test, one-way analysis of variance, Mann-Whitney U, Kruskal-Wallis tests, multiple linear regression models.	experienced moderate to high moral distress during the COVID-19 pandemic. Moral distress and emotional exhaustion were strongly correlated.
Sajjadi et al. (2017)<br>Canada	Cross-sectional study	40 internal medicine residents.	To examine the relationship between moral stress and burnout in medical residents.	Self-reported questionnaires<br><br>Moral Distress Scale Revised (Hamric et al., 2012)<br>alpha = NR<br><br>Emotional Exhaustion from MBI (Maslach & Jackson, 1986)<br>alpha = NR	Descriptive statistics, unpaired t-test, analysis of variance, Pearson correlations, Wilcoxon signed rank sum test.	Medical residents experienced moral distress, with significant differences during intensive care units (ICU) and clinical teaching units (CTU) rotations. Moral distress was correlated with emotional exhaustion.
Kellish et al. (2021)<br>USA	Cross-sectional study	75 health science clinical educators (health science professionals, occupational and physical therapists, speech language pathologists, social workers)	To determine if a relationship exists between the moral distress and burnout in health science clinical educators.	Self-reported questionnaires<br><br>Moral Distress Scale-Revised (Hamric et al., 2012).<br>alpha = NR<br><br>Emotional Exhaustion from MBI (Maslach & Jackson, 1986)<br>alpha = NR	Descriptive statistics, Pearson correlations, one-way ANOVA, post-hoc analyses using Bonferroni multiple comparison tests.	Particular personality traits appear to play a role in the moral distress and burnout experienced by clinical educators. A significant correlation existed between burnout and emotional exhaustion.

Kok et al. (2023)<br>The Netherlands	Cross-sectional study	251 intensive care unit (ICU) professionals (nurses, physicians) from two Dutch hospitals.	To disentangle the associations of ICU professionals' moral distress and other risk factors with the components of burnout	Self-reported questionnaires<br><br>Moral Distress Scale-Revised (Hamric et al., 2012)<br>alpha = NR<br><br>Emotional Exhaustion from MBI (Maslach & Jackson, 1986)<br>alpha = NR	Descriptive statistics, independent t-tests, non-parametrical Mann-Whitney U test, multivariate regression models, mediation models, moderation models.	Levels of moral distress were similar in physicians and ICU nurses. Moral distress was significantly related to emotional exhaustion.
Grasso et al. (2022)<br>Italy	Cross-sectional study	Healthcare providers (nurses, physicians) from pediatric intensive care unit (PICU).	To develop and validate the pediatric version of the Italian version of the Moral Distress Scale-Revised.	Self-reported questionnaires<br><br>Moral Distress Scale-Revised (Hamric et al., 2012).<br>alpha = .87<br><br>Emotional Exhaustion from MBI (Maslach & Jackson, 1986)<br>alpha = NR	Descriptive statistics, correlations, reliability analysis, factor analysis.	Nurses showed a higher moral distress score then physicians. The relationship between moral distress and burnout was not significant.
Christodoulou-Fella et al. (2017)<br>Cyprus	Cross-sectional study	206 mental health nurses from public hospitals.	To explore the association among moral distress, secondary traumatic stress syndrome, and emotional exhaustion.	Self-reported questionnaires<br><br>Moral Distress Scale-Revised (Hamric et al., 2012)<br>alpha = NR<br><br>Emotional Exhaustion.<br>One question "Please, indicate from 1 to 10	Descriptive statistics, t-test and one-way ANOVA, correlations, linear regression models.	A significant relationship between moral distress and emotional exhaustion was observed. Emotional exhaustion mediate the association between moral distress and secondary traumatic stress syndrome.

				how emotionally exhausted do you feel due to your work?" alpha = NR		
Fumis et al. (2017) Brazil	Cross-sectional study	283 critical care unit providers (physicians, nurses, nurse technicians, respiratory therapists).	To estimate both burnout syndrome and moral distress prevalence among critical care unit providers and to estimate the correlation between moral distress and burnout.	Self-reported questionnaires<br><br>Moral Distress Scale-Revised (Hamric et al., 2012)<br>alpha = NR<br><br>Emotional Exhaustion from MBI (Maslach & Jackson, 1986)<br>alpha = NR	Descriptive statistics, one-way ANOVA, correlations, logistic models.	Severe burnout syndrome was present in all critical care providers, specially in nurses and respiratory therapists. A positive relationship between moral distress and emotional exhaustion was observed.
Meltzer y Huckabay (2004) USA	Cross-sectional study	60 critical care nurses of two public hospitals.	To determine the relationship between critical care nurses' perceptions of moral distress, futile care and burnout.	Self-reported questionnaires<br><br>Moral Distress Scale-Revised (Corley, 1995)<br>alpha = .95<br><br>Emotional Exhaustion from MBI (Maslach & Jackson, 1986)<br>alpha = NR	Descriptive statistics, t-tests, post hoc Scheffe tests, correlations, linear regression models.	A significant relationship between moral distress and emotional exhaustion was observed.
Ohnishi et al. (2010) Japan	Cross-sectional study	391 psychiatric nurses.	To develop an moral distress scales for psychiatric nurses and to explore the relationship between moral	Self-reported questionnaires<br><br>Moral Distress Scale-Psychiatric (Ohnishi et al., 2010).<br>alpha = 0.90	Descriptive statistics, factor analysis, correlations, multiple linear regression models.	A significant correlation was demonstrated between moral distress and emotional exhaustion. The ratio of patients to nurses was found to influence exhaustion.

			distress and burnout.	Emotional Exhaustion from MBI (Maslach & Jackson, 1986)<br>alpha = NR		

Notes
NR: Not reported
